# Coelomic Fluid Evaluation in *Pisaster ochraceus* Affected by Sea Star Wasting Syndrome: Evidence of Osmodysregulation, Calcium Homeostasis Derangement, and Coelomocyte Responses

**DOI:** 10.3389/fvets.2020.00131

**Published:** 2020-03-06

**Authors:** Sarah J. Wahltinez, Alisa L. Newton, Craig A. Harms, Lesanna L. Lahner, Nicole I. Stacy

**Affiliations:** ^1^Department of Comparative, Diagnostic, and Population Medicine, College of Veterinary Medicine, University of Florida, Gainesville, FL, United States; ^2^Wildlife Conservation Society, Bronx, NY, United States; ^3^Department of Clinical Sciences and Center for Marine Sciences and Technology, College of Veterinary Medicine, North Carolina State University, Morehead, NC, United States; ^4^Seattle Aquarium, Seattle, WA, United States

**Keywords:** chemistry, cytology, echinoderm disease, invertebrate, ochre sea star, osmolality

## Abstract

Sea Star Wasting Syndrome (SSWS) is one of the largest marine wildlife die-offs ever recorded, killing millions of sea stars from more than 20 Asteroid species from Alaska to Mexico from 2013 to 2015 from yet undetermined cause(s). Coelomic fluid surrounds the sea star's organs, playing critical roles in numerous systemic processes, including nutrient transportation and immune functions. Coelomocytes, which are cellular components of coelomic fluid and considered functionally equivalent to vertebrate leukocytes, are responsible for innate cell-mediated immunity. The objectives of this study were to (1) evaluate changes in coelomic fluid chemistry, coelomocyte counts, and cytology from ochre sea stars (*Pisaster ochraceus*) (*n* = 55) with clinical signs consistent with SSWS at varying intensity (SSWS score 1: *n* = 4, score 2: *n* = 2, score 3: *n* = 3, score 4: *n* = 18, score 5: *n* = 26) in comparison to coelomic fluid from clinically normal sea stars (*n* = 26) and to (2) correlate SSWS score with cellular and biochemical analytes. SSWS-affected sea stars had wider ranges of all electrolytes, except calcium; statistically significantly higher chloride, osmolality, and total protein; lower calcium; and higher coelomocyte counts when compared to clinically normal sea stars maintained under identical environmental conditions. Free and/or phagocytized bacteria were noted in 29% (16 of 55) coelomic fluid samples from SSWS-affected sea stars but were absent in clinically normal sea stars. SSWS score correlated significantly with increasing chloride concentration, osmolality, and coelomocyte counts. These chemistry and cytological findings in coelomic fluid of SSWS-affected sea stars provide insight into the pathophysiology of SSWS as these results suggest osmo- and calcium dysregulation, coelomocyte responses, and presumptive opportunistic bacterial infection in SSWS-affected sea stars. This information provides potential future research applications for the development of treatment strategies for sea stars in managed care and for understanding the complexity of various biochemical and cellular pathophysiological mechanisms involved in sea star wasting.

## Introduction

Sea star wasting or mass stranding events have been reported since the 1960s along the North American Pacific (1, 2) and Atlantic Coasts (3–6) as well as the Mediterranean Sea (7), Atlantic coast of Europe (8), and East China Sea (9). These historical wasting events were limited in geographic scope and impacted only a few species. Sea Star Wasting Syndrome (SSWS) is one of the largest recorded marine wildlife die-offs, resulting in death of millions of sea stars from more than 20 species from 2013 until 2015 (10). Sea Star Wasting Syndrome was first noted off the coast of the Pacific Northwest in mid-2013 and later impacted sea stars from Southern Alaska to Baja California, Mexico (11, 12). The ochre sea star (*Pisaster ochraceus*), the keystone species of the rocky intertidal zone (13), was among the most heavily affected species. The resulting collapse of sea star populations caused a trophic cascade with large scale, complex effects on marine invertebrate population dynamics along the Pacific Coast (14).

Sea Star Wasting Syndrome impacted sea stars begin to show behavioral changes of abnormally curled rays and inability to grasp substrate, progressing to exhibit white epidermal lesions, ray autotomy, loss of turgor (deflation), and loss of structural integrity leading to disintegration and eventual death (11, 12). The progression of clinical observations can be rapid, typically leading to death within days (15). Histologically, wasting sea stars consistently exhibit epidermal degeneration, necrosis, and ulceration with dermal separation, necrosis, and inflammation (16). The etiology for these histological changes has not been determined to date.

There is evidence that marine echinoderm mass mortality events may be associated with pathogens and/or environmental stressors and it is presumed that various underlying etiologies or inciting causes may result in similar clinical signs characteristic for SSWS (10). For this reason, we have chosen to use the terminology Sea Star Wasting Syndrome rather than Sea Star Wasting Disease as it was initially referred to in previous publications by various authors (11, 12). Due to limited types of tissue reactions to pathogens and other stressors in echinoderms, various disease processes reportedly result in similar clinical and histopathological manifestations (16). The ultimate cause(s) of the 2013–2015 SSWS event as well as most historical and more recent observations of sea star wasting in various geographical areas are still unknown. No infectious causes were definitively linked to SSWS, although a densovirus and a novel circular DNA virus were isolated from *Pycnopodia helianthoides* (11) and *Asterias forbesi* (17), respectively. Associations between increased ocean temperature and clinical signs of wasting have been identified for *P. helianthoides* (18), *P. ochraceus* (19, 20), and *Astropecten johnstoni* (7). In contrast to those reports, wasting of *P. ochraceus* along the Oregon coast was linked to decreased temperature (12), but no clear association between temperature and SSWS was found based on an analysis of 20 years of data from 88 geographical sites (15).

Coelomic fluid fills the coelomic cavity and surrounds the internal organs of asteroid sea stars, so it can provide information on systemic biochemical and cellular processes and potential response mechanisms to disease. Coelomocytes, the invertebrate counterpart to vertebrate leukocytes, are an integral part of the sea star immune system and circulate in coelomic fluid. They are responsible for cellular innate immune defense through interactions with the complement system, cytokines, lectins, antimicrobial peptides, proteins, clotting factors, and protein mediators that facilitate cellular adhesion (21–23). Sea stars are osmoconformers when placed in hypertonic and hypotonic sea water, equilibrating inorganic ions within 24 h (24). Sea stars are isoionic with seawater and appear capable of minimally maintaining ionic homeostasis for potassium and calcium (25–27). They are able to regulate their coelomic fluid volume through unknown mechanisms (28, 29).

While coelomocyte concentrations (30–32) and chemistry data (24, 32) have been published for clinically normal sea stars, no comparisons between SSWS affected and clinically normal sea stars have been published to date. The objectives of this study were to (1) evaluate changes in coelomic fluid chemistry, coelomocyte counts, and cytology from ochre sea stars (*n* = 55) with clinical signs consistent with SSWS at varying intensity score on a grading scale of 1–5 in comparison to coelomic fluid from clinically normal sea stars (*n* = 26) and to (2) correlate SSWS score with cellular and biochemical analytes.

## Materials and Methods

Coast-inhabiting ochre sea stars were collected from Puget Sound ranging from several days to several weeks prior to this study during spring 2015. Sea stars were categorized as early-adult if their arm radius was 8–10 cm and late-adult if their arm radius was >10 cm. The sea stars were clinically normal at time of collection and the entire cohort of these collected sea stars began to show signs consistent with SSWS once under managed care. This was expected and considered a progression of disease in these animals collected from areas knowingly affected by this syndrome. The occurrence and progression of clinical signs observed for these sea stars was consistent with those concurrently reported for free-ranging sea stars (20). The sea stars were adults of unknown age at time of coelomic fluid sampling when showing clinical evidence of SSWS in June and July 2015. The sea stars were maintained in several well-established, natural sea water flow-through quarantine tanks with an average temperature 10°C (50°F) and average pH 7.8 and species-appropriate husbandry (33) at the Seattle Aquarium, an Association of Zoo and Aquariums-accredited facility. Water quality parameters, including salinity, were measured at least every 7 days. Clinically normal sea stars (*n* = 26) that were collected several years before SSWS in the area and well-established at the aquarium were maintained in a similar manner but in display tanks separate from the sea stars with clinical evidence of wasting (32).

Sea stars were scored at the time of coelomic fluid sampling on a SSWS grading scale of 1–5 adapted from a scoring system proposed by Bates et al. (19) as briefly outlined in the following and Table 1. Grade 0 sea stars were clinically normal on visual and physical examination and displayed appropriate activity and feeding behaviors. Grade 1 sea stars had mild disease without white lesions. These sea stars often displayed central disc flattening, lack of adherence to substrate, mild swelling of arms, and abnormal wrapping of arms at rest. Grade 2 sea stars had mild disease with small white lesions. These lesions were restricted to one arm or only the central disc. Grade 3 sea stars had moderate disease with white lesions found on one arm plus the central disc or two arms or an arm/disc interface. Grade 4 sea stars had severe disease. These sea stars had lesions found on three or more arms or two arms plus central disc or arm/disc interface plus arm or central disc or more than one arm/disc interface lesion. Grade 5 sea stars had severe disease with one or more arms detached from the central disc or the sea star was found unresponsive, unattached to the substrate and with no tube foot movement.

**Table 1:** Clinical observations based on a 1–5 grading scale in sea stars affected by Sea Star Wasting Syndrome, adapted from Bates et al. (19).

**Score**	**Description**
0	Clinically normal
1	Mild disease, no white lesions; often central disc flattening, lack of adherence to substrate, mild swelling of arms, and abnormal wrapping of arms at rest
2	Mild disease with small white lesions restricted to one arm or only the central disc
3	Moderate disease with white lesions on one arm plus the central disc OR two arms OR an arm/disc interface
4	Severe disease; white lesions on three or more arms OR two arms plus central disc OR arm/disc interface plus arm or central disc OR more than one arm/disc interface lesion
5	Severe disease; one or more arms detached from the central disc OR non-responsive, detached from substrate and lacking tube foot movement

Native coelomic fluid was collected from the perivisceral coelomic cavity using a 23-ga. 2.5 cm needle and syringe ~1 cm from the distal tip of a ray on the aboral surface of the sea star. A total volume of at least 1.0 ml was collected per individual. Prior to performing necropsies of deceased sea stars (SSWS score 5), all collectable coelomic fluid was aspirated. Coelomic fluid was transferred into aliquots with and without additives as outlined in the following.

Samples of 200 μl native, undiluted coelomic fluid were transferred into lithium heparin microtubes and analyzed for magnesium, sodium, potassium, chloride, calcium, and total protein (Ortho Vitros 250 analyzer, Ortho Clinical Diagnostics, Rochester, New York 14626 USA). Osmolality was measured in coelomic fluid aliquots without any additives or anticoagulants using a freezing point depression osmometer (Advanced Instruments Osmometer, model 3320, Norwood, MA) at Cornell University's Animal Health Diagnostic Center.

After coelomic fluid collection, direct smears were immediately prepared for dry mount cytology using native coelomic fluid. Coelomic fluid was placed in both lithium heparin and ethylenediaminetetraacetic acid (EDTA) micro tubes (Sarstedt Inc., Princeton, NJ). Formalin preparations of 50 μl coelomic fluid in 200 μl 10% neutral buffered formalin and 50 μl EDTA coelomic fluid in 200 μl 10% neutral buffered formalin were prepared for coelomocyte counts for comparison (34). Sediment smears were prepared after centrifugation of 200 μl of coelomic fluid at 605 relative centrifugal force (RCF) for 10 min, drawing off the supernatant, re-suspending the pellet in 50 μl supernatant, and preparation of smears from this concentrated cellular material for dry mount cytology. Direct and sediment dry mount smears were air-dried and stained with Wright-Giemsa (Harleco®, EMD Millipore, Billerica, Massachusetts, USA) for cytological evaluation by one investigator who was blinded to animal ID and SSWS score. Manual coelomocyte counts were performed on native (non-EDTA preserved) and EDTA-anticoagulated formalin fixed preparations as described for lobster hemolymph and fish blood in due consideration of the dilution factor resulting from formalin dilution (34).

Statistical analyses were performed in the programming language Python (Version 3.7.3) using the numerical library SciPy (Version 1.3.0). The full source code is freely available for review at https://bit.ly/2OmrMt1. To determine if there was a statistically significant difference in salinity between different systems and sampling dates, a Kruskal Wallis H-test (one-way ANOVA on ranks) was performed as this data did not meet the assumption of normality. The assumption of normality was only met in clinically normal sea stars for magnesium, sodium, potassium, chloride, and calcium (32) and for sodium, chloride and calcium in SSWS-affected sea stars per D'Agostino and Pearson's test. To determine if there was a statistically significant difference (*P* ≤ 0.05) between the distributions of tested analytes, osmolality, and coelomocyte counts from SSWS-affected sea stars and those from clinically normal sea stars, a Welch's *t*-test was selected for analytes that met the assumption of normality and a non-parametric Kruskal-Wallis H-test for those that did not. A paired *t*-test was used to evaluate if there was a difference in cell counts between formalin preparations with EDTA and those with native coelomic fluid. To evaluate the correlation between SSWS-score and analytes, a Spearman's rank-order correlation coefficient was computed and its statistical significance was determined using the t-distribution.

## Results

Coelomic fluid was collected from 55 SSWS-affected early-adult (*n* = 22) and late-adult (*n* = 33) sea stars. The following SSWS scores were identified: score 1: *n* = 4, score 2: *n* = 2, score 3: *n* = 3, score 4: *n* = 18, score 5: *n* = 26. The distribution of sea stars among SSWS scores represented the rapid disease progression (typically within several days) and severity observed in the collection.

### Coelomic Fluid Chemistry

**Figure 1:**
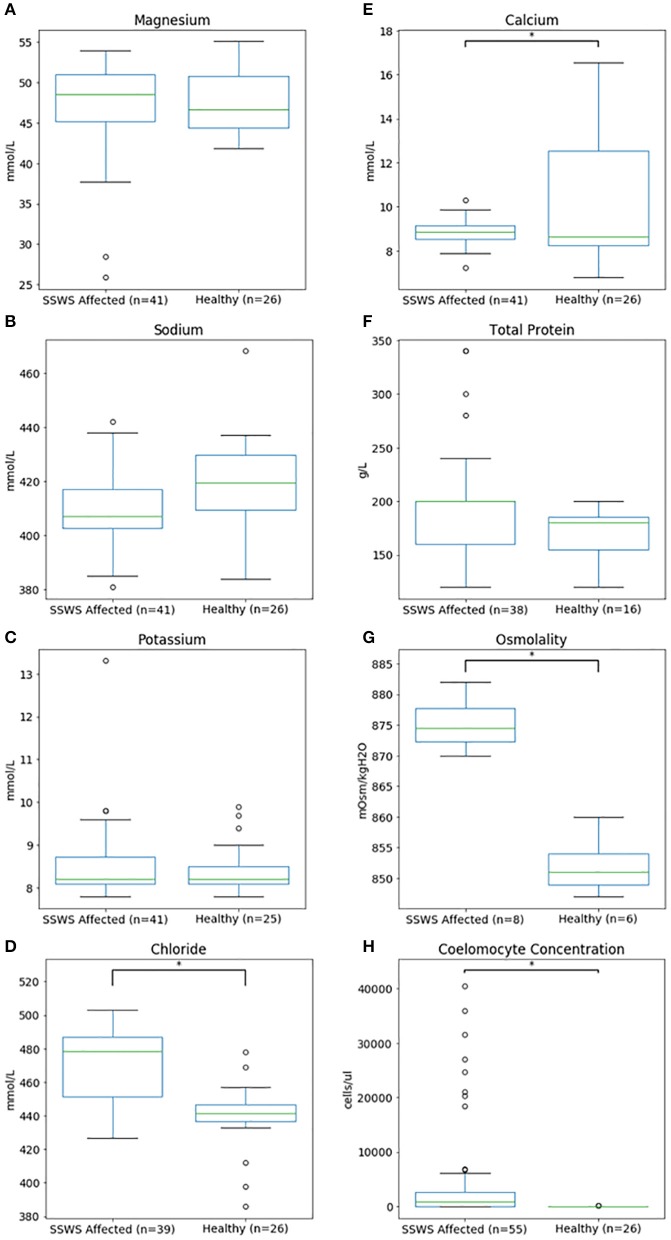
Box plots comparing coelomic fluid chemistry data **(A–F)**, osmolality **(G)**, and coelomocyte counts **(H)** of *Pisaster ochraceus* showing clinical signs consistent with Sea Star Wasting Syndrome compared to clinically normal *P. ochraceus*. *Denotes statistical significance (*P* < 0.05).

Biochemical analysis was performed of coelomic fluid from 41 and of osmolality from 8 SSWS-affected sea stars (Figure 1). Diseased sea stars demonstrated wider ranges of all chemistry data than clinically normal sea stars, except for calcium. SSWS-affected sea stars had wider ranges of electrolytes, with statistically significantly higher chloride (*P* = 1.3 × 10^−7^). Total protein (*P* = 0.03) was significantly higher and calcium (*P* = 0.048) significantly lower when compared to clinically normal sea stars. SSWS-affected sea stars (*n* = 8) had higher osmolality (*P* = 3.4 × 10^−3^) than clinically normal sea stars (*n* = 6). SSWS-affected sea star coelomic fluid chemistry and coelomocyte count data are presented in Table 2.

**Table 2:** Coelomic fluid chemistry data and coelomocyte counts of *Pisaster ochraceus* showing clinical signs consistent with Sea Star Wasting Syndrome.

**Analyte**	**Units**	***n***	**Mean**	**SD**	**Median**	**Range**	90% CI^*^
Magnesium	mmol/L	41	46	7.5	48	16–54	43–53
Sodium	mmol/L	41	412	15.8	407	381–463	396–442
Potassium	mmol/L	41	8.8	1.9	8.2	7.8–19.1	8.0–9.0
Chloride	mmol/L	39	473	25.7	479	427–553	416–461
Calcium	mmol/L	41	8.7	1.0	8.9	4.2–10.3	7.4–13.7
Total Protein	g/L	38	21	69.6	20	<10–50	Range: <10–20
Coelomocytes	^#^/μl	55	6126	12393.1	900	0–60,750	Range: 0–180
Osmolality	mOsm/kgH_2_O	8	875	4.3	875	870–882	NA

CI, Confidence Interval for clinically normal sea stars*; NA, not available.

* *Wahltinez et al. (32)*.

# *Number of cells*.

### Coelomocyte Counts

Coelomocyte counts of SSWS-affected sea stars were highly variable, ranged from 0 to 60,750/μl (median: 900; mean: 6,126), and had significantly higher coelomocyte counts (*P* = 1.5 × 10^−7^) compared to clinically normal sea stars (median: 0; mean: 13; Table 2). Both formalin preparations (with and without EDTA) were consistent with each other across all samples for cell counts (*P* = 0.36). Several (*n* = 15, 27%) SSWS-affected sea stars had 0/μl coelomocyte counts; their SSWS scores were score 1: *n* = 3; score 2: *n* = 1; score 3: *n* = 2; score 4: *n* = 7; score 5: *n* = 2.

### Cytological Evaluation

**Figure 2:**
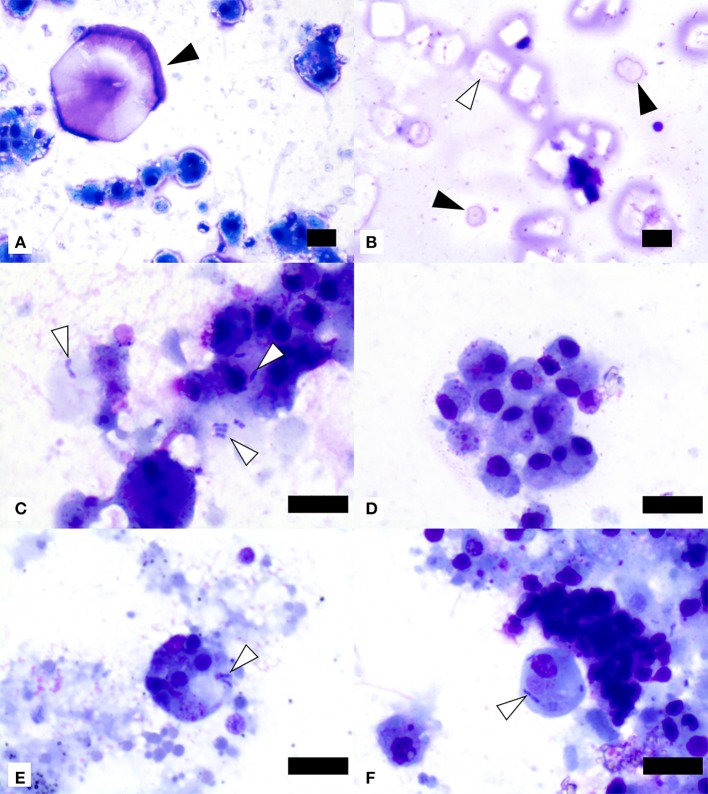
Image composite of coelomic fluid sediment smear preparations from ochre sea stars (*Pisaster ochraceus*) affected by Sea Star Wasting Syndrome. **(A)** Presumptive salt crystal (black arrowhead) and mononuclear phagocyte morphotype cells showing variable vacuolation. **(B)** Presumptive cholesterol crystals (one example shown by white arrowhead) and two presumptive salt crystals (black arrowheads). **(C)** Extracellular and phagocytized bacilli (white arrowheads) in close association with mononuclear phagocyte morphotype cells. **(D)** Mononuclear phagocyte morphotype cells with variable amounts of phagocytized material of undetermined origin. **(E,F)** Two mononuclear phagocyte morphotype cells with phagocytized small bacilli (white arrowheads). Scale bars = 10 μm.

Cytological evaluation was performed on all 55 SSWS-affected sea stars. There was consistently one coelomocyte type present with morphology of the mononuclear phagocyte morphotype based on previous descriptions of coelomocyte morphology (23, 31). The coelomocytes were vacuolated in 10 samples (18%) and/or had phagocytized pink or basophilic material of undetermined origin in 32 samples (58%). Free (*n* = 3), phagocytized (*n* = 5), or both free and phagocytized (*n* = 8) bacteria were present in 16 samples (29%). The bacteria were predominantly plump or bipolar bacilli (*n* = 14), coccobacilli with concurrent bacilli (*n* = 1), or coccobacilli with concurrent bacilli and diplococci (*n* = 1). Unknown extracellular basophilic material was noted in 20 samples (36%) and material suggestive of proteinaceous origin or nucleoproteinaceous necrotic debris in 36 samples (65%). There were frequently lysed and disintegrated cells in 35 samples (64%). Crystals suggestive of cholesterol crystals were seen in 9 samples (16%), crystals suggestive of salt origin in 17 samples (31%), and mineral material in 7 samples (13%). Representative images of cytological findings are included in Figure 2.

Clinically normal sea stars reportedly have the same predominant coelomocyte morphotype suggestive of the mononuclear phagocyte morphotype, extracellular crystals suggestive of salt origin, and no bacteria or other microorganisms (32).

### Sea Star Wasting Syndrome Score

**Figure 3:**
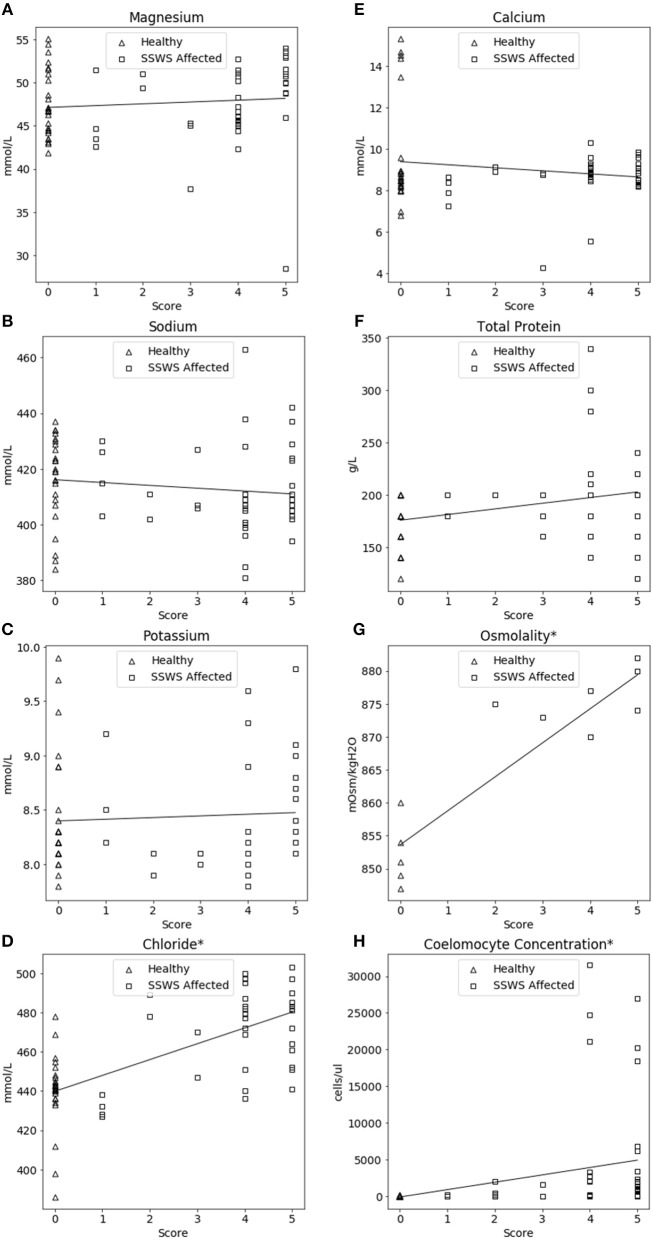
Correlation plots comparing coelomic fluid chemistry data **(A–F)**, osmolality **(G)**, and coelomocyte counts **(H)** of *Pisaster ochraceus* showing clinical signs consistent with Sea Star Wasting Syndrome (SSWS) compared with clinically normal *P. ochraceus* grouped by SSWS Score (0–5). *Denotes statistical significance (*P* < 0.05).

SSWS score was significantly correlated with higher chloride concentration (rho = 0.63, *P* = 2.44 × 10^−8^), coelomocytes (rho = 0.67, *P* = 1.54 × 10^−11^), and osmolality (rho = 0.85, *P* = 2.54 × 10^−4^). The association between SSWS score and analytes is included in Figure 3.

### Tank Water Salinity

Salinity was maintained between 29 and 32 ppt during the sampling period. There was no significant difference in salinity between different systems and sampling dates (*P* = 0.12), including comparisons of tank systems of SSWS-affected seas stars and those holding clinically normal sea stars.

## Discussion

This is the first report of comprehensive coelomic fluid analysis in SSWS-affected sea stars showing evidence of biochemical and cellular alterations pointing toward potential underlying pathophysiological processes associated with the syndrome.

### Dysregulation of Critical Biochemical Regulatory Processes Are Presumptively Involved in Sea Star Wasting Syndrome Pathophysiology

The significant increases in chloride, total protein, and osmolality suggest osmodysregulation. The increase in coelomic fluid osmolality may indicate that SSWS-affected sea stars are actively concentrating one or more active osmolytes; additional considerations include that energy-dependent cross-membranal transport mechanisms and/or pumps may be affected by wasting, or that protein may leak into the coelom due to cellular apoptosis and/or necrosis. If sea stars are truly becoming hyperosmotic to seawater by a yet unknown mechanism or cumulative mechanisms, this could explain why relatively hypoosmotic seawater could penetrate tissues and cause biochemical homoeostatic imbalances as seen in SSWS. Histopathology of sea stars with clinical signs consistent with SSWS showed extensive necrosis, epidermal ulceration, and dermal separation (16). In a study with ochre sea stars exposed to changes in salinity, coelomic fluid chloride concentrations approximated those of seawater, thus suggesting sea stars may not actively regulate chloride homeostasis (27). Although the sample size tested for osmolality was limited, our results provide evidence for osmodysregulation in sea stars affected by SSWS and an incentive for further study of osmoregulatory pathways.

Season and reproductive cycle stage have been associated with effects on the ionic regulation in *Asterias* (25, 35, 36). Season is unlikely to have contributed to the differences described in this study as coelomic fluid from SSWS and clinically normal sea stars was sampled in the boreal summer. Sea stars with clinical signs consistent with SSWS were often noted to spawn in later stages of wasting. Spawning may occur as a terminal behavior in response to electrolyte changes or other stressors.

Mutable collagenous tissue (MCT) is unique to echinoderms and capable of rapid, neuronal-mediated changes in tensile strength that are not due to muscle fibers (37–39). In sea stars, MCT has been found in the body wall (40, 41), in ligaments at spine joints, and in the stem of tube feet (42). The effect of ion concentrations on MCT variable tensility has been studied extensively on *in-vitro* preparations, primarily of holothurian dermis. Samples of MCT placed in solutions with higher calcium concentrations display stiffening, while MCT samples placed in solutions with decreased calcium concentrations result in softening (43–48). The SSWS-affected sea stars with significantly lower coelomic fluid calcium concentrations all displayed softening of the body wall and lesions of the integument, suggesting MCT involvement in SSWS progression. However, clinically normal sea stars had wider ranges of coelomic fluid calcium concentrations, including some calcium values lower than the SSWS-affected sea stars, and all clinically normal sea stars displayed normal MCT stiffness. Given the altered calcium regulation in SSWS-affected sea stars in this study, further studies could investigate the effect of ion concentrations on MCT *in-vivo*, how ion channels impact MCT mutability and integrity, and mechanisms for changes to ion concentrations in coelomic fluid.

### Sea Star Wasting Syndrome-Affected Sea Stars Show Active Cellular Immune Responses

The observed higher coelomocyte counts in SSWS sea stars suggest active cellular immune responses similar to studies that have demonstrated that environmental stressors and disease can result in increased circulating coelomocytes in echinoderms (30, 49, 50). The lack of differences in coelomocyte counts in EDTA- and heparin-preserved samples indicate that either anticoagulant can be used. At this time, it is unknown whether the use of anticoagulants is necessary for coelomic fluid analysis and if native coelomic fluid can be used for coelomocyte counts. Further study is warranted.

Several (*n* = 15, 27%) SSWS-affected sea stars had coelomocyte counts of 0/μl which was inconsistent with increased coelomocyte numbers observed in the majority of SSWS-affected sea stars. This may represent an artificial decrease due to cellular clumping or aggregation (51); however, cell clumping was only noted in 12 samples with counts above 810/μl, none of which had a zero coelomocyte count. In addition, the observed low coelomocyte counts in SSWS-affected sea stars may have resulted from individual variation in disease progression or an individual's inability to mount an effective immune response due to external factors resulting in immunocompromise. Previous studies have shown that temperature, salinity, and/or parasitism impact coelomocyte response and sea star immunity, although none of these factors were noted in the sea stars in the current study (30).

The free and phagocytized bacteria presumptively represent secondary infection due to disturbance(s) in body wall integrity as they were not identified in the majority of SSWS coelomic fluid samples, but in none from unaffected sea stars. Culture of coelomic fluid was beyond the scope of this study and not attempted. The basophilic and proteinaceous material as well as the presumptive cholesterol crystals noted on cytology are probably due to apoptosis or necrosis as described by meta-transcriptomic analysis (52) and histopathology (16) in SSWS-affected sea stars.

### Sea Star Wasting Syndrome Severity Correlates With Biochemical and Cellular Changes

The significant correlations between SSWS score with chloride, osmolality, and coelomocyte counts are consistent with the direct comparison of SSWS-affected and clinically normal sea stars. However, due to the low sample size for osmolality those results should be interpreted cautiously. The SSWS score appears to have a predictive ability with chloride, osmolality, and coelomocyte counts and reflect severity of clinical disease.

## Conclusions

The results of this study provide guidance for future studies of sea star osmo- and calcium regulatory pathways and cellular immune functions. This study provides information on chemical and basic cellular components in coelomic fluid of SSWS- affected sea stars, furthering our understanding of the pathophysiology of this devastating syndrome. Evaluation of calcium, chloride, total protein, osmolality, and coelomocyte counts in individuals or a cohort of sea stars, in comparison with the biochemical composition of their surrounding water, may provide an earlier diagnosis of onset of disease and advanced disease states. By understanding that osmoregulation is impacted by SSWS, therapeutic targets and interventions may be created to stop or reverse disease progression in individuals under managed care. The evidence of an opportunistic bacterial infection suggests why there are anecdotal reports of SSWS-affected sea stars responding to antimicrobial therapy but failing to respond in other cases. Treating these secondary infections may allow individual sea stars to more effectively compensate for the primary problem. Enrofloxacin has been shown to reach therapeutic levels via intracoelomic injection (53) and may be useful depending on antimicrobial sensitivity testing. Furthermore, the application of the information provided herein provide insight into potential complex pathophysiological mechanisms of SSWS and lays the groundwork for future research strategies relevant to the conservation of sea stars as keystone species in marine ecosystems.

## Data Availability Statement

The datasets generated for this study are available on request to the corresponding author.

## Ethics Statement

Although Institutional Animal Care and Use Committee review is not required for non-cephalopod invertebrates, this study was conducted in adherence to general ethical principles.

## Author Contributions

LL and AN were responsible for study design. SW collected coelomic fluid samples with the oversight of LL. SW and NS drafted the manuscript. All authors assisted in writing and editing of the manuscript and performed data analysis and interpretation.

### Conflict of Interest

The authors declare that this study received funding from Boeing Company. The funder was not involved in the study design, collection, analysis, interpretation of data, the writing of this article or the decision to submit it for publication.
